# Barrier Impairment and Type 2 Inflammation in Allergic Diseases: The Pediatric Perspective

**DOI:** 10.3390/children8121165

**Published:** 2021-12-09

**Authors:** Michele Ghezzi, Elena Pozzi, Luisa Abbattista, Luisa Lonoce, Gian Vincenzo Zuccotti, Enza D’Auria

**Affiliations:** 1 Allergology and Pneumology Unit, V. Buzzi Children’s Hospital, 20154 Milan, Italy; enza.dauria@unimi.it; 2 Department of Pediatrics, V. Buzzi Children’s Hospital, 20154 Milan, Italy; elena.pozzi@asst-fbf-sacco.it (E.P.); luisa.abbattista@unimi.it (L.A.); luisa.lonoce@unimi.it (L.L.); gianvincenzo.zuccotti@unimi.it (G.V.Z.); 3 Department of Biomedical and Clinical Science “L. Sacco”, University of Milan, 20157 Milan, Italy

**Keywords:** children, inflammation, biologics, severe asthma, allergic rhinitis, atopic dermatitis, eosinophilic esophagitis

## Abstract

Allergic diseases represent a global burden. Although the patho-physiological mechanisms are still poorly understood, epithelial barrier dysfunction and Th2 inflammatory response play a pivotal role. Barrier dysfunction, characterized by a loss of differentiation, reduced junctional integrity, and altered innate defence, underpins the pathogenesis of allergic diseases. Epithelial barrier impairment may be a potential therapeutic target for new treatment strategies Up now, monoclonal antibodies and new molecules targeting specific pathways of the immune response have been developed, and others are under investigation, both for adult and paediatric populations, which are affected by atopic dermatitis (AD), asthma, allergic rhinitis (AR), chronic rhinosinusitis with nasal polyps (CRSwNP), or eosinophilic esophagitis (EoE). In children affected by severe asthma biologics targeting IgE, IL-5 and against IL-4 and IL-13 receptors are already available, and they have also been applied in CRSwNP. In severe AD Dupilumab, a biologic which inhibits both IL-4 and IL-13, the most important cytokines involved in inflammation response, has been approved for treatment of patients over 12 years. While a biological approach has already shown great efficacy on the treatment of severe atopic conditions, early intervention to restore epithelial barrier integrity, and function may prevent the inflammatory response and the development of the atopic march.

## 1. Introduction

Allergic diseases are a global burden in terms of health-care resources and on patients’ quality of life. They include a broad spectrum of diseases, affecting airways, the skin, and the gastrointestinal tract, and they present with different clinical manifestations. Patients with one allergic disease have a higher probability of having other atopic comorbidities, suggesting that these conditions share common pathways [1,2].

In 2017, Pothoven and Schleimer hypothesized that epithelial barrier dysfunction can result in the development of allergic diseases, firstly proposing the ‘barrier hypothesis’ for type 2 inflammatory diseases [3].

This hypothesis has been recently revisited by C. Akdis, who argued that the impairment of the epithelial barrier, caused by different damaging agents linked to industrialization, urbanization, and modern life, may explain the rise in allergic, autoimmune and other chronic conditions: the so-called extended ‘epithelial barrier hypothesis’ [4].

The dysfunction of the epithelial barriers in different body sites is characterized by an impairment in cellular differentiation, junctional integrity, and innate defence. Genetic predisposition, environmental factors, and abnormal inflammatory cascades can contribute to the establishment and maintenance of the epithelial barrier dysfunction, leading to exposure to environmental and food allergens with consequent allergic sensitization and chronic disease [5]. Several allergens, pathogens and environmental agents, including cigarette smoke, surfactants, enzymes and emulsifiers, present in processed food nanoparticles and microplastics, can damage the epithelial barrier [6,7,8].

Allergen exposure can trigger an abnormal type 2 immunity response, leading to the recruitment of inflammatory cells in barrier sites with consequent tissue inflammation, disruption, and remodelling. At first, antigens penetrate through the damaged barrier, then they trigger the activation of an innate immune response involving epithelial cells and resident immune cells. In this phase, antigens are captured by dendritic cells (DC) and presented to naive T cells, inducing their differentiation into T helper 2 (Th2) cells. These Th2 cells favour the B cells’ production of a specific isotype of immunoglobulin (IgE), and its receptor is expressed by mast cells and basophils. In this phase, a pool of memory B and Th2 cells is created [9].

Further exposure to the same antigens causes cross-linking between antigens and IgE bound-to-mast cells and leads to the immediate activation of an immune cascade, which is rapid, amplified and effective. Collaterally, this enormous inflammatory response causes tissue damage and a subsequent repair process, leading to tissue remodelling.

It has been long debated whether inflammation is the first cause of barrier dysfunction or if the epithelial barrier impairment can promote allergen exposure and then trigger an abnormal inflammatory response.

**Figure 1:**
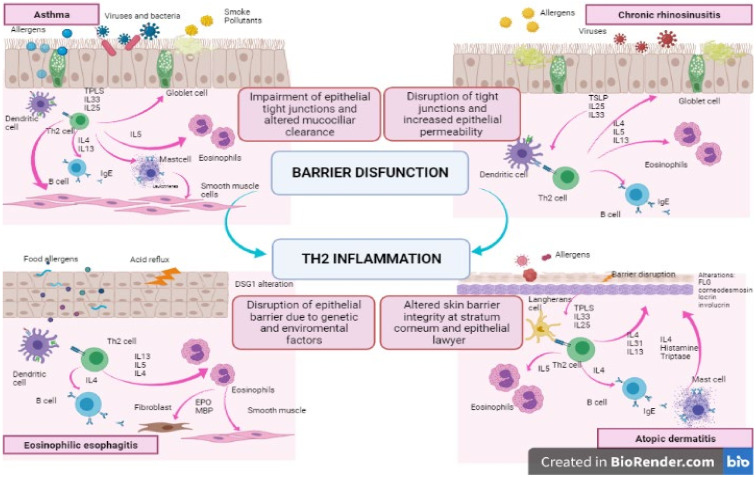
Barrier dysfunction and type 2 inflammatory response in asthma, CRSwNP, AD, and EoE.

On the one hand, an abnormal immune response at barrier sites, due to repeated allergen exposure, causes perpetual damage to epithelial integrity and to the consequent repair process, inducing a vicious circle of injury-repair (see Figure 1). Conversely, recent studies suggest that a pre-existing barrier dysfunction may be demonstrated in most atopic patients before the appearance of allergic manifestations. Furthermore, mouse models with defective epithelial homeostasis have showed spontaneous allergic sensitisation. New evidence supporting the primary role of epithelial damage in the pathogenesis and progression of allergic diseases has been growing [3].

The alterations in the composition of the microbiota, e.g., a reduced biodiversity, have been demonstrated in different allergic diseases [10,11,12,13]. The status of dysbiosis provokes tissue inflammation due to an up-regulation of pro-inflammatory environmental, which, in turn, promotes barrier damage, leading to a vicious circle.

This review aims to give an overview of the pathogenetic role of barrier dysfunction in allergic diseases in children. We hereby focus on atopic dermatitis (AD), asthma, allergic rhinitis (AR), chronic rhinosinusitis with nasal polyps (CRSwNP), and eosinophilic esophagitis (EoE). Understanding the central pathways involved in the establishment of allergic diseases may be essential in developing new strategies for their prevention and treatment.

## 2. Methods

This review focuses on epithelial barrier dysfunction and type 2 inflammatory response in asthma, allergic rhinitis, chronic rhinosinusitis, atopic dermatitis, and eosinophilic esophagitis. A comprehensive search was conducted using the electronic databases MEDLINE via PubMed, Embase databases, and Web of Science. The keywords used were: asthma, allergic rhinitis, chronic rhinosinusitis, atopic dermatitis, eosinophilic esophagitis, epithelial barrier dysfunction, type 2 inflammatory diseases, and children.

## 3. Barrier Dysfunction in Asthma: How Tight Junctions and Altered Mucus Play an Important Role

Asthma is a chronic inflammatory disease of the airways, characterized by an airflow obstruction. It’s the most common chronic respiratory disease in childhood, and despite improvement in its care, asthma remains a significant public health problem [14].

The typical symptoms are coughing, wheezing, shortness of breath, and chest tightness. They occur episodically due to a sudden, but reversible, airway constriction (an acute inflammation on a chronic basis). Exacerbation factors include viral infections, exposure to allergens and irritants, exercise, emotion, change in weather, and humidity.

Recent insights highlight the important role of the airway epithelium as one of the main factors involved in the development of, but also the maintenance of, asthma.

The airway epithelium is one of the barriers protecting our body from external noxious agents. In healthy patients, it prevents the penetration of inhaled pathogens such as allergens, pollution, viruses, bacteria, and fungi [15].

Normally, the epithelium is pseudostratified in the trachea and bronchi and cuboidal in the bronchioles. This cellular barrier is covered with mucus that contains antimicrobials peptides and antibodies that trap and transport inhaled particles to the mouth where they can be swallowed or expectorated [16,17].

In asthma, both these physical barriers are impaired due to endogenous genetic variations and to chronic inflammation processes.

To begin, asthmatic patients exhibit both quantitative and qualitative alteration of mucociliary clearance (MCC). On the one hand, mucin content is increased between 8–15% (normal range 2%). This abnormal amount of mucin changes the quality of mucus, leading to an impaired function of the cilia [17,18].

On the other hand, we can find an alteration in the ratio of MUC5AC to MUC5B. Normally, the mucus of healthy individuals predominantly contains MUC5B. In asthmatic patients, we find a reduced amount of MUC5B and an increased expression of MUC5AC [19].

This unbalanced process is primarily due to the genetic alteration in the expression of MUC5AC, KIF3A, and EFHC1 [20], and it causes mucus overproduction, airway obstruction, and the accumulation of undesired substances, resulting in chronic inflammation.

Notably, patients with type Th2-high signature asthma consistently show an increased production of MUC5AC,—a process mediated by both IL-13 and epidermal growth factor.

Secondly, it is due to the epithelial barrier efficiency in the three types of junctions that anchor a cell to its neighbours. These junctions are: the Adherens junction (AJs), the Tight junction (TJs), and hemidesmosomes. AJs form zonula adherens, in which different components are involved (e.g., E-cadherin, actinin, vinculin, alfa-catenin, and beta-catenin). TJs form a complex called zona occludens that contains claudins, occludins, and junctional adhesion molecules. In healthy airways, TJs and AJs constitute a dense network that prevent the movement of basically all molecules.

In asthmatic patients, we typically find a lack of, and a dysfunction of, E-cadherin [21], alfa-catenin, ZO-1, and occludin [22], leading to an impaired barrier function. This alteration is primarily due to a genetic abnormality in different genes such as PCDH1 and CDHR3 [23,24]. Moreover, viral infections induce epithelial alarmins (IL-25, IL-33, e TSLP), which cause the activation of type-2 innate lymphoid cells (ILC2) [25,26,27].

These cytokines, with a variety of direct and indirect mechanisms, interact with Th2 immunity cells and stimulate the production of typical Th2 cytokines (IL-4, IL-5, IL-13) inducing, once again, a barrier dysfunction [28,29,30]. In turn, genetic abnormalities cause an overexpression of alarmins, which represents a strong risk factor for the development of asthma [31,32].

However, not only genetics impact barrier integrity and function. Infections may also induce epithelial dysfunction through three different mechanisms: viruses cause a modification of the junctions’ function; they also destroy epithelial cells due to their cytotoxic and cytopathic effect; lastly, the antiviral immune response releases Th2-type cytokines that directly affect epithelial barrier integrity, inducing a downregulation of E-cadherin, beta-catenin, and occluding [33,34]. In addition, allergens, captured by dendritic cells (DCs), activate Th2 cells that produce cytokines such as IL-4, IL-5, and IL-13. These Th2 cytokines drive IgE production by B-lymphocytes (IL-4), eosinophilic infiltration into the airways (IL-5), goblet cell hyperplasia, and excessive mucus hypersecretion (IL-13), causing a significant barrier dysfunction, that, in turn, leads into an increased susceptibility towards pathogens and allergens.

Last but not least, allergens also promote the spread of the inflammatory process and may contribute to airway remodelling [35].

Another pathogenetic mechanism that has been hypothesized as playing a role in barrier dysfunction is the mesenchymal transition of the epithelial cells, due to infections and loss of tight junctions causing a reduction in cell repair capacity and cell differentiation [36].

To sum up, chronic exposure to allergens and respiratory viruses causes serious damage to the barrier, inducing dysfunction and disruption of the epithelial barrier. All these mechanisms increase the barrier’s permeability, creating a vicious circle between inflammation and barrier damage. “Which came first?” is a question that currently remains unanswered.

## 4. Therapeutic Implications and Novel Treatment Strategies

Taking all the above into consideration, we can certainly consider the epithelial barrier of the airways as a potential therapeutic target for new treatment strategies, in particular with the aim of strengthening TJ and AJ and controlling mucus production.

**Figure 2:**
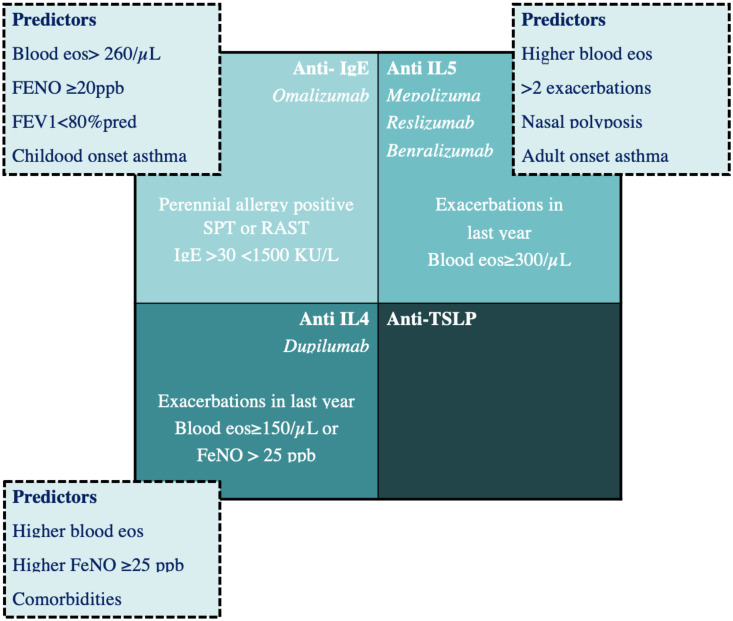
Biologics approved for the treatment of severe asthma: mechanisms, patient selection criteria, and predictors of good responses.

Over the last decades, new monoclonal antibodies to bind specific targets of the immune response, both for the adult and the paediatric population, have been developed. This has markedly changed the therapeutic approach: in paediatric asthma, this novelty was initially represented by Omalizumab, a recombinant DNA-derived humanized anti-IgE monoclonal antibody [37], but subsequently, other biologics have been approved for use and others are under investigation (see Figure 2).

Omalizumab is indicated as an add-on treatment for children with severe allergic asthma with elevated serum IgE (>30 and <1500 IU/mL) and serum IgE positivity for at least one aeroallergen. After binding circulating IgE, Omalizumab decreases IgE levels, inhibits IgE binding with its receptors, and downregulates the expression of high-affinity IgE receptors FcεRI on mast cells, basophils, and dendritic cells [38]. Overall, this results in a decreased release of inflammatory mediators related to the allergic response.

Decreased sputum, bronchial eosinophils and T cells were observed in adult bronchial biopsies after Omalizumab [37]. Omalizumab has been highlighted as having a possible role in reducing the expression of mediators of tissue remodelling at the bronchial level [39].

The efficacy and safety of Omalizumab in the paediatric population emerged from several RCTs [40,41,42].

It was demonstrated that Omalizumab reduces the number and frequency of exacerbations when withdrawing ICS and also improves quality of life [41].

Its efficacy in reducing seasonal exacerbations, triggered by respiratory viruses, has also been reported. The mechanism, not yet fully understood, probably involves the restoration of antiviral defences (for example, type I interferon production) [43].

Despite the long-term clinical experience in the use of Omalizumab, the optimal duration of therapy in patients who achieved a good clinical response, and the long-term effects after discontinuation, still need to be defined. The efficacy and safety of Omalizumab, even 24 months after its suspension, have recently been demonstrated [44].

Mepolizumab is an anti-IL-5 humanized monoclonal antibody that reduces circulating eosinophils.

Mepolizumab has demonstrated a favourable efficacy profile in decreasing the number of asthma exacerbations, improving lung function, controlling asthma, and quality-of-life (QoL) scores, as well as significantly reducing OCS use [45,46,47].

Mepolizumab has shown a good pharmacokinetic, pharmacodynamic, and safety profile, both in the short and long term, even in the paediatric population between 6 and 11 years, and it has also demonstrated efficacy in improving asthma control [48].

Mepolizumab is currently indicated as an adjunct treatment of severe eosinophilic asthma (>150 cells/μL) in adults, and in children over 6 years of age.

Reslizumab, an anti-IL-5 humanized monoclonal antibody, has been approved by the European Medicines Agency as an add-on therapy in adults with uncontrolled severe eosinophilic asthma (blood eosinophil count ≥400 cells/μL). It is the only drug to be administered intravenously, with the dosage based on the patient’s weight. It decreases exacerbations and improves lung function and quality life [49]. Benralizumab binds both the IL-5 receptor subunit and the FcγRIIIa receptor, expressed in natural killer cells, inducing a rapid depletion of eosinophils through a mechanism of apoptosis. Benralizumab has been effective in reducing asthma exacerbations and the use of OCS in adults [50]. Currently, Benralizumab is approved in adults (>18 years) with severe eosinophilic asthma.

Dupilumab is a human monoclonal antibody effective against the IL-4 receptor chain (IL-4Ra) blocking downstream signalling via both the IL-4 and IL-13 receptors.

Dupilumab is approved for the treatment of severe type 2 asthma characterized by high levels of serum eosinophils and/or FeNO.

Dupilumab has been effective in reducing both asthma exacerbations and improving pulmonary function in 107 adolescents (>12 years) with severe asthma [51]. Dupilumab reduces steroid doses in adults and adolescents [52].

Recently, a phase III trial (NCT02948959), aimed at evaluating the efficacy of Dupilumab in children aged 6 to 12 years with uncontrolled persistent asthma, was concluded [53,54], as well as a phase III trial in children aged 6 to 11 affected by severe atopic dermatitis.

There are not enough data on validated biomarkers that predict responses to different available biological therapies in children. Recent studies have demonstrated that Omalizumab is more effective in asthmatic children with comorbidities (multiple sensitizations, atopic dermatitis, and food allergies), with high peripheral eosinophil counts and with a high pre-treatment total IgE and high fractional exhaled nitric oxide levels [55]. Further investigation is needed to highlight the presence of predictors of good responses to specific pharmacological therapies, which, in turn, could be useful in applying personalized therapies (see Figure 2).

Moreover, the identification of phenotypic heterogeneity, especially in severe asthma, in both adults and children, has stimulated the research for phenotype-specific interventions towards precision medicine [56].

Eosinophilic or allergic asthma phenotypes represent the target for biologics, while there are no approved therapeutic strategies specific for patients with confirmed eosinophil-low asthma.

An alternative approach may be to target an upstream mediator of the inflammatory response in order to achieve effective asthma control in different endotypes of severe asthma. A potential target is thymic stromal lymphopoietin (TSLP), which is activated by multiple triggers, such as viruses and other irritants. Tezepelumab is a first-in-class human monoclonal antibody that blocks the activity of TSLP acting, in part, by inhibiting the production of pathologic mucins. Clinical trials with Tezepelumab have showed promising results in patients with a variety of asthma phenotypes, demonstrating significant reductions in exacerbations and improvements in lung function, symptom control, and HRQoL. Phase 3 trials of Tezepelumab are underway with the aim of assessing the potential efficacy of Tezepelumab in patients with a broad range of severe asthma phenotypes, evaluating both the oral corticosteroid-sparing potential of Tezepelumab and the effect of Tezepelumab on airway inflammation and airway remodelling [57]. Monoclonal antibodies targeting IL-33 or ST2 are in clinical development, in phase 2 trials (see Table 1). No clinical studies of anti-IL-25 antibodies are currently in progress, although the potential role in virus-induced asthma exacerbations [58].

## 5. Barrier Dysfunction in Allergic Rhinitis and Chronic Rhinosinusitis: The Important Role of Mucus Hypersecretion and Goblet Cells Up-Regulation

Allergic Rhinitis (AR) is an IgE-mediated inflammatory disorder of the nasal mucosa induced by allergens.

It’s one of the most common chronic diseases in children and has a considerable impact on quality of life. Its incidence depends on age and domicile, and its prevalence is globally increasing [59].

Clinically, AR is characterized by four major symptoms: rhinorrhoea, sneezing, nasal itching, and nasal obstruction [60].

In this case, recent studies highlight the essential role of the nasal epithelium in the development and progression of AR [61].

The nasal epithelium plays a key role in protecting the body. It is often the first tissue to come into contact with allergens, pollutants, pathogens, and other noxious stimuli.

In healthy patients, the first third of the nasal cavity is formed by a stratified squamous epithelium lying over a layer of proliferative cells. The further two thirds of the cavity are lined with a pseudostratified columnar ciliated epithelium, which contains goblet cells which, in turn, overlie a basement membrane.

Even in AR, this physical barrier is impaired due to genetic variations and chronic inflammation.

AR is characterized by an impaired nasal epithelial barrier integrity due to dysfunction and disruption of the Tight Junction (TJ).

Normally nasal epithelial cells are anchored to one other by Tjs, which form a complex called zona occludens (ZO) containing claudins, occludins, and junctional adhesion molecules [62]. These proteins are essential in regulating the passage of ions and molecules through the membrane [63].

In patients with AR, we can detect a modified gene expression, which causes a reduction in ZO-1 [64].

Moreover, the allergens’ protease can disrupt the tight Junctions (specifically ZO-1). This was evident specifically for Dermatophagoides pteronyssinus and various pollens. The mechanisms involved are numerous, but they all directly damage TJ proteins increasing epithelium permeability [65,66].

Finally, the Th2-inflammatory process releases specific cytokines such as IL-4, IL-5, and IL-13 which directly affect the epithelial barrier inducing a disruption of ZO-1 [64].

In AR, the primum movens is the allergic response. The development of allergic sensitization that characterizes AR is determined by a strong genetic component; in fact, atopic patients inherit the predisposition to develop immune responses IgE/mast cells/TH2 lymphocytes [67].

The allergen-driven inflammatory response is led by IgE overproduction causing, among other mediators, the release of histamine. The synthesis of IgE is driven by the exaggerated Th2-response and the dysfunction of T-regulatory cell 1 response [68]. Moreover, Th2 inflammation is responsible for the release of cytokines (IL-4 and IL-13), the recruitment, maturation, and survival of accessory cells such as eosinophils, basophils, and mast cells.

All these mechanisms (especially histamine and cytokine IL-4 and IL-13 production) are crucial in altering the epithelial permeability interfering with the expression of TJs [69].

Moreover IL-4, IL-5, and IL-13 induce chronic inflammation creating a self-maintaining vicious circle.

Chronic Rhinosinusitis with Nasal Polyps (CRSwNP) is a particular phenotype of chronic rhinosinusitis (CRS) based on endoscopy and computed tomography findings of nasal polyposis. The diagnosis requires four cardinal symptoms: nasal obstruction, drainage, loss of smell, and facial pain or pressure that persist over 3 months [70].

It is an undeniable burden for healthcare even if an accurate measure of its incidence has yet to be determined [71].

As well as in asthma, abnormalities in the physical barrier, mucociliary clearance, and local innate antimicrobial responses have been described in CRSwNP. The latter is characterized by an irregular and decreased expression of the TJ molecules such as occludin and ZO-1 and weakened desmosomal junctions [72,73]. Moreover, TJ integrity is negatively affected by inflammation and viral infections [74]. All these factors combine to induce a functional and structural dysfunction of the epithelial barrier.

CRSwNP patients present a quantitative and qualitative alteration in mucus production due to the alteration of pendrin, periostin, and PLUNC family molecules [75]. This results in an inefficient clearance of the nasal airways and in the accumulation of foreign antigens contributing to chronic inflammation.

Several external pathogens such as bacteria (especially S. Aureus), fungi, viruses, and allergens induce nasal epithelial cells to produce epithelial derived cytokines (TSLP, IL-33 and IL-1). Each of these cytokines is able to activate, via both innate and adaptive immunity, type 2 inflammation. On the one hand TSLP induces Th2 cell differentiation causing the activation of adaptive type 2 inflammation. On the other hand, TSLP and IL-33 stimulate ILC2s to produce type 2 cytokines (IL-4, IL-5, IL-13) via an innate type 2 inflammation. Finally, TSLP, IL-33, and IL-1, stimulating epithelial and mucosal mast cells, produce IL-5 and IL-13 [76,77,78].

Type-2 cytokines, following the same pathways of asthma, induce IgE production by B-cells and plasma cells (IL-4), eosinophilic infiltration into the airways (IL-5), goblet cell hyperplasia and excessive mucus secretion (IL-13) building the foundation for chronic inflammation and subsequent tissue remodelling [79].

## 6. Therapeutic Implications and Novel Therapeutic Strategies

In this case, improving the integrity and function of the nasal epithelial barrier represents the mainstay therapeutic strategy.

The easiest way to preserve barrier efficiency is to control the inflammatory response. To this purpose, antihistamine drugs and topical glucocorticoids have already been used in clinical practice as a first line treatment strategy. However, some patients with moderate-to-severe AR have an inadequate response to currently recommended medications [80]. In particular, in this group, the use of biological therapies, such as Omalizumab, could be life-changing. Moreover, particular benefits could be derived from asthmatic patients with comorbidity [81,82].

However, the use of Omalizumab for the treatment of AR has not yet been approved neither in the adult nor in the paediatric population.

Instead, the use of a biological therapy has already proven its efficacy in adult patients suffering from CRSwNP and could be of undoubtable interest in the paediatric population as well.

Omalizumab was the first biological drug approved for treatment in adult patients (18 years and over) with CRSwNP, following the same dosage schedule used in the treatment of severe asthma. Three phase III trials (NCT03280537, NCT03280550, NCT03478930) showed that patients treated with Omalizumab achieved a significant reduction in respiratory symptoms and nasal polyp size as early as after only 4 weeks of treatment [83].

Moreover, Dupilumab has recently been approved for the treatment of CRSwNP, as several trials have demonstrated its efficacy and safety in adult patients [53]. It led to a significant reduction in the endoscopic nasal polyp score in CRSwNP, but it also showed improvement in the self-reported sense of smell in patients without asthma. Furthermore, 58% of the population studied had undergone prior nasal surgery, suggesting a possible target patient [84]. These data suggest a possible major role of signalling pathways mediated by IL-4 and IL- 13 in the pathogenesis of CRSwNP.

Three studies reported the use of anti–IL-5 treatment, and they showed greater improvement in the endoscopic nasal polyp score in the treatment groups [85,86,87]. In the Reslizumab study, the findings were statistically significant in those patients with high intranasal IL-5 levels. Gavaert et al. showed that Mepolizumab is effective in reducing symptoms and polyp size in adults with steroid-refractory CRSwNP [88]. In two phase III trials (NCT03085797, NCT03401229), Mepolizumab and Reslizumab were, respectively, safe and effective as an add-on therapy for bilateral CRSwNP [80]. Paediatric studies are not currently available.

## 7. Barrier Dysfunction in Atopic Dermatitis: The Pivotal Role of Stratum Corneum

The skin is an important immunological organ that acts as a primary barrier between the body and the environment. It consists of epidermal proteins of the stratum corneum (SC), stratum granulosum, tight junctions, and epidermal lipids, such as ceramides. The SC is crucial for skin barrier function. It is the outer layer of skin, placed above the epithelial layer of keratinocytes and interspersed antigen-presenting cells called Langerhans cells (LC) [89].

The SC is composed of proteins, including filaggrin, involucrin, loricin, etc., and an outer lipid layer. Keratin filaments are aggregated through filaggrin (FLG) monomers which derive from pro-filaggrin cleavage. FLG degradation products contribute to the natural moisturizing of the SC and to the maintenance of the acid pH of the epidermidis [90,91,92]. The extracellular matrix of the SC is composed of lipids such as ceramides, long-chain fatty acids, and cholesterol that play essential roles in maintaining epidermal permeability and guaranteeing skin barrier integrity [93,94].

There has been increasing evidence in the last decades that barrier impairment is the hallmark of AD [95].

Epidermal barrier dysfunction results in increased permeability, reduced integrity of the epidermis, increased transepidermal water loss (TEWL), drying of the skin, and ruptures of the skin [96]. Of note, skin barrier impairment has been observed in lesioned skin but also in skin without lesions [97].

In infancy, the skin barrier is physiologically more permeable due to low lipid concentration in the SC and the reduced production of FLG cleavage derivates. In particular, FLG products are lower in cheeks than in elbow or nasal tips during the first year of life and this may explain why cheeks are a common first site of AD in early childhood [98,99].

A number of different genes have been implicated in the pathogenesis of AD, provoking structural abnormalities of the epidermis and immune dysregulation [100].

Several studies have demonstrated that FLG loss-of-function mutations are associated with atopic eczema [101,102,103] and AD phenotype in FLG null-mutation has an earlier onset, greater severity, and increased persistence [104]. Furthermore, inflammatory cytokines produced in AD, especially IL-4 and IL-13, have been demonstrated to reduce FLG expression in keratinocytes, leading to the perpetuation of a vicious circle of inflammation and tissue damage [105,106,107].

However, nearly 40% of patients with FLG null-alleles don’t exhibit the AD phenotype, suggesting that pathophysiology of AD goes far beyond FLG mutations [92].

In fact, other proteins of the corneal layer such as corneodesmosin, locrin, and involucrin can be altered in AD with the consequent impairment of barrier skin function and type-2 immune activation [104,108,109,110]. Additionally, lipids such as ceramides are diminished in patients with AD and inflammatory cytokines released during allergic responses can further reduce the lipid content of the SC and worsen barrier dysfunction [93,111,112,113].

Environmental factors such as detergents and microplastics have been demonstrated to alter skin barrier integrity. In particular, anionic surfactants in commercial detergents can disrupt the tight junctions of the SC [6] while other components, such as papain, promote the release of inflammatory cytokines [114].

The impairment of barrier function facilitates the penetration of allergens and microbial pathogens that can trigger an unwanted immune response to innocuous environmental *stimuli* leading to AD and/or other systemic allergies [89].

The importance of skin barrier impairment as *primum movens* of the pathogenetic mechanism is supported by the observation that skin barrier dysfunction, in the first week of life, detected as increased transepidermal water loss, has been associated with the increased risk of developing allergies in the first 2 years of life [99,115].

Finally, evidence from past decades has shown that skin microbiome plays a crucial role in the maintenance of cutaneous homeostasis and the defence against pathogenic microorganisms. AD is characterized by a dysregulation of microbiome which can be caused by many environmental factors such as pH, temperature, dryness, hygiene practices, and antibiotics; all these factors can alter the richness and the diversity of resident bacteria with notable consequences for skin homeostasis [116].

Coagulase-negative staphylococci (CoNS) such as Staphylococcus epidermidis and Staphylococcus hominis are among the most common Gram-positive species inhabiting the human skin [117]. These bacteria play an active role in contrasting the colonization of Staphylococcus aureus and other pathogens [118]. The lack of antimicrobial peptides in AD and disorders of innate immunity including Toll-like receptors favour virulent strains of Staphylococcus aureus colonization [119].

The dysbiosis characterizing AD skin, with a higher colonization of Staphylococcus aureus, can alter the SC protein and lipid composition, and it can exacerbate skin inflammation by Th2 cytokines [93,118,120,121,122].

In general, the immune response activated by penetrating agents includes a first sensitization phase and a subsequent effector one. The sensitization phase is initiated when allergens are captured by LC and transported to local lymphonodes. There, antigen presentation to naive CD4+ T cells drives the proliferation and differentiation of them into Th2 cells (allergen specific CD4 T cell), releasing high levels of cytokines as IL-4 and IL-13. In the presence of these cytokines, B-cells are driven to produce specific IgE and to generate a memory pool of allergen-specific B cells and CD4+ positive Th2 cells [107].

Epithelial cells in AD skin play a role in initiating the immune cascade during the sensitization phase through the secretion of thymic stromal lymphopoietin (TSLP) that can activate DCs and stimulate them to migrate to skin draining lymphonodes where they promote CD4+ differentiation. TSLP is highly expressed by keratinocytes in the SC of patients with AD and by epithelial cells in asthmatic children [123], and it seems to be correlated with AD severity in children valuated by SCORAD [124]. Elevated expression of TSLP can be found in the skin months before the development of AD [125]. Of note, TSLP promotes migration of skin presenting-antigens to mesenteric lymphonodes creating a sort of skin to gut migration [107,126].

IL-33 is another important cytokine released and involved in inflammatory cascade in AD [107,127]. Epithelial cells physiologically produce IL-33, which resides in the cellular nucleus where it controls gene expression. In the event of barrier cell disruption, IL-33 is released and acts as an alarm signal by binding to mast-cells, DCs, resident macrophages, and group 2 innate lymphoid cells, inducing the production of inflammatory cytokines and proteases to digest connective tissue and favour leukocyte penetration [127]. IL-33 deficient mice have a reduced severity of food-induced reactions, suggesting a potential role of this cytokine in food allergies.

Gain of function genes encoding these cytokines and their receptors can contribute to development of AD [128,129,130]. Interestingly, not only can hereditary mutations alter gene expression, but also epigenetic mechanisms may regulate cytokine production. Epigenetic modifications, such as genomic DNA modification and microRNA posttranscriptional regulation can be inherited or acquired through environmental exposure. In particular, stress, obesity, low vitamin D levels, and poly-aromatic hydrocarbon exposure due to fuel or tobacco combustion can lead to an increased expression of specific cytokines [131,132,133].

The second phase of the immune response is called the effector phase and is triggered by a second contact between the sensitised host and the allergen. Antigen-presentation activates memory allergen-specific Th cells that produce IL-13, IL-4, and IL-5. These cytokines promote recruitment of mast-cells with specific receptors for IgE that are activated and produce more cytokines. High levels of allergen specific IgE are maintained and inflammatory cells, such as eosinophils, are recruited to the inflamed site with consequent tissue damage.

The pathogenesis of AD cannot be exclusively explained through Th2 activation. In fact, prominent Th17 activation has been observed in the blood of AD patients. IL-17 produced by Th17 can reduce FLG and involucrin expression and contribute to skin barrier dysfunction [93,134,135]. Serum IL-17 is positively correlated with total IgE levels in patients, with AD suggesting a role of this cytokine not only in AD but also in other allergic phenotypes [136].

Recent studies have shown that a group of skin resident cells, called group 2 innate lymphoid cells (ILC2), may play an important role in allergic immune responses. These cells are dispersed through barrier surfaces of the skin, gut, and airways, and they can secrete pro-allergen cytokines as IL-5 and IL1-3, which, in turn, promote the recruitment of inflammatory cells, alteration in skin microbiome, and dysfunction of the skin-barrier [93,94,95,96,97,98,99,100,101,102,103,104,105,106,107], perpetuating a vicious circle.

## 8. AD and the Atopic March

In the light of this evidence, skin barrier damage in AD may be the first step of the so-called “atopic march”, a clinical sequence beginning with AD and culminating with food and respiratory allergies [93]. Animal models have demonstrated that exposure to food allergens after epicutaneous barrier disruption can induce an increase in specific Ig, respiratory, and gastrointestinal allergic symptoms [89,93,136,137].

In fact, mechanical skin injury in AD causes a systemic release of IL-33, leading to the activation of intestinal mast cells and an increase in intestinal permeability, promoting anaphylaxis in patients with AD and food allergies [138,139].

Similarly, IL-17 and IL-23 levels in children with AD have been correlated with association of AD and other atopic diseases, suggesting their role as markers of the atopic march [140].

Age and severity at the onset of AD are positively correlated with the risk of developing other allergies in the future. Children with AD, requiring topical steroids in the first 3 months of life, have a 50.8% possibility of developing a challenge-proved food-allergy [141] and there seems to be a dose-dependent correlation between food sensitizations and SCORing topic Dermatitis (SCORAD) levels in children between 4 and 11 months of age [142]. Furthermore, early-onset (<6 months) AD is associated with the increased risk of respiratory allergies in childhood.

Overall, this evidence highlights that the early treatment of AD skin can have a potential role in the prevention of the atopic march. Evidence has suggested that skin barrier improvement, via the daily application of emollients, beginning in the neonatal period in high-risk infants, reduces the risk of AD development [143,144].

Several preliminary studies have proved that AD can be prevented through skin-barrier improvement intervention. Although these trials have showed that regular moisturizing can prevent AD, it is still unclear whether AD prevention is achieved, or AD is merely delayed or masked. Furthermore, there aren’t any data demonstrating the long-term effects of the application of emollients on the risk of AD beyond the treatment period and on the incidence of food allergies [93].

## 9. Novel Therapeutic Strategies

Coinciding with a deeper understanding of AD pathogenesis, many new therapies have been developed, and others are under investigation (see Table 1). Biologics and small molecules, targeting different metabolic pathways (see Table 1), represent the new frontiers in the treatment of AD.

Nowadays, the so-called TH2 immune pathway has been recognized as an important mechanism involved in many inflammatory reactions, underpinning different allergic conditions [145]. The Th2 immune pathway starts in the skin at the site of sensitization and ultimately leads to the inflammatory reaction.

IL-4 and IL-13 are the two most important cytokines involved in Th2 inflammatory pathway of AD [146,147]. Recent evidence has also suggested that the shared receptor subunit for IL-4 and IL-13 (IL-4Ra) on sensory neurons can mediate chronic pruritus through Janus kinase (JAK) signalling.

Dupilumab, a biologic targeting IL-4Ra, inhibits both IL-4 and IL-13 signalling [148,149].

In adolescents with moderate-to-severe AD, Dupilumab has shown clinically meaningful and statistically significant improvements in AD signs and symptoms, including pruritus [150].

Other therapeutic strategies under investigation aim to block, specifically, either IL-4 or IL-13 and other cytokines involved in inflammatory pathways, such as IL-5, IL-31, and IL-22.

Blocking IL-33 through monoclonal antibodies seems to be a promising therapy not only for AD but also for preventing food allergy development and the progression of the atopic march [151,152].

Current studies are investigating the protein and lipid barrier deficits of early AD and will guide future preventative interventions, but these studies imply the need for the early prediction of AD and act prophylactically as early as the neonatal period.

In addition to these strategies, manipulating skin microbiota in the first period of life is under investigation. Colonization, with commensal staphylococci at 2 months of age, has been associated with a lower risk of developing AD at 1 year of age [153], suggesting the potential role of the topical application of commensal bacteria as a preventive measure.

**Table 1:** Biologic treatment in atopic dermatitis (AD), asthma, chronic rhinosinusitis with nasal polyps (CRSwNP), or eosinophilic esophagitis (EoE).

Disease	Biologic Drug	Action	Indication	Age of Use	Stage of Experimentation
ASTHMA	Omalizumab	anti-IgE	severe allergic asthma with elevated serum IgE (>30 and <1500 IU/mL) and serum IgE positivity for at least one aeroallergen	-	IV-Approved
	Mepolizumab	anti-IL5	severe eosinophilic asthma	>6 yo	IV-Approved
	Reslizumab	anti-IL5	severe eosinophilic asthma (blood eosinophil count ≥400 cells/μL)	-	IV-Approved
	Benralizumab	anti-IL5	severe eosinophilic asthma	>18 yo	IV-Approved
	Dupilumab	anti-IL4	severe eosinophilic asthma	>12 yo	IV-Approved III-6–12 yo—Paller A.S. et al. [54]
	Tezepelumab	anti-TSLP	variety of asthma phenotypes	-	III-Menzies-Gow A. et al. [57]
CRSwNP	Omalizumab	anti-IgE	CRSwNP with asthma	>18 yo	IV-Approved
	Dupilumab	anti-IL4	CRSwNP	>18 yo	IV-Approved
	Mepolizumab	anti-IL5	CRSwNP with steroid-refractory	>18 yo	III-Gavaert et al., Licari et al. [80,86]
	Reslizumab	anti-IL5	CRSwNP	>18 yo	III-Licari et al. [80]
EoE	Dupilumab	anti-IL4	EoE	>18 yo	II-Hirano et al. [154]
				1–12 yo	III-recruitment
AD	Dupilumab	anti IL4 e IL13	moderate to severe atopic dermatitis	>12 yo	
	Baricitinib	anti JAK 1 e 2	Moderate to severe DA	>18 yo	IV-Approved EMA 2019
	Tralokinumab	anti IL13	Moderate to severe DA	>18 yo	IV-Approved EMA 2021
	Abrocitinib	anti JAK 1	Moderate to severe DA	>2 yo	IV-Approved EMA 2020

## 10. Barrier Dysfunction in Eosinophilic Esophagitis: The Interplay between Impairment of Epithelial Barrier Integrity and Inflammatory Response

Eosinophilic Esophagitis (EoE) is an immune-mediated chronic disease characterized by symptoms related to oesophageal dysfunction and by the presence of eosinophil-predominant oesophageal inflammation [155,156]. EoE is histologically defined as more than 15 eosinophils per high power field in an oesophageal mucosal biopsy.

Since it was first reported, the incidence and prevalence of EoE have been increasing over time, with similar rates in both children and adults [157]. Clinical manifestations of EoE vary by age group and are not pathognomonic. Infants and children commonly present with feeding difficulties, vomiting, and failure to thrive, while heartburn, dysphagia, and food impaction have been found to be more common in adolescents [158].

It is now believed that EoE occurs in genetically predisposed individuals in whom food allergens penetrate through a deficient epithelial barrier and trigger an abnormal immune reaction mediated by Th2 cytokines, leading to oesophageal lesions, dysmotility, subsequent remodelling, and fibrosis [159].

Multiple evidence supports the theory that EoE is closely related to atopy. Most EoE patients have allergic comorbidities or a family history of atopy [160]. It is known that an Elemental Diet can induce both clinical and histological remission in EoE patients [161]. Moreover, EoE lesions can be induced in experimental models by allergen exposure through the skin, respiratory, or gastrointestinal systems [162,163,164,165].

Nevertheless, EoE pathogenesis appears to be more complex than a mere Ig-E mediated condition. Numerous evidence supports a mixed IgE-mediated and delayed Th2-mechanism in EoE pathogenesis. In fact, IgE and B cell deficient mice can experimentally develop EoE [155] and monoclonal anti-IgE antibodies that failed to induce remission in EoE patients [166].

The genetic contribution of EoE to aetiology is suggested by the increased risk of developing the disease in first degree relatives or siblings of EoE patients [167,168]. An insight into the genetic contribution to EoE is given by mono-genetic inherited disorders with an increased risk of EoE development. Dysregulation of TGF-ß signalling, a key cytokine involved in epithelial growth, fibrosis, and tissue remodelling, is thought to be the pathogenic mechanism for the development of EoE in inherited connective tissue disorders (CTD) (e.g., Loetyz–Dietz Syndrome, Marfan Syndrome type II and Ehlers–Danlos Syndrome) [169], while the gene mutation involved in epithelial integrity and tissue remodelling (e.g., STAT3, DSG1, SPINK5) can explain the increased risk of EoE in patients affected by PTEN Hamartoma Tumour Syndrome, SAM (severe skin dermatitis, multiple allergies and metabolic wasting) Syndrome, Netherton Syndrome, and Autosomal Dominant Hyper-IgE Syndrome [109,170,171,172]. This evidence suggests the pivotal role of gene involvement in mucosal barrier integrity, inflammatory cell recruitment, and tissue remodelling in EoE pathogenesis. Furthermore, analysis of EoE transcriptome, a panel of 574 genes, has demonstrated a different pattern of gene expression involved in mucosal barrier integrity and inflammatory response, in the oesophagus of EoE patients, healthy controls, and patients with chronic esophagitis [173,174,175].

Specifically, the epidermal differentiation complex (EDC), a gene complex encoding for proteins, such as desmosomal cadherin desmoglein-1 (DSG1), fillaggrin, and involucrin, involved in barrier integrity, has been demonstrated to be markedly downregulated in EoE tissues [176]. Consistently, DSG1 expression is decreased in EoE biopsies, but not in gastroesophageal reflux disease (GERD) biopsies [177].

The deficiency of epithelial barrier integrity can be histologically demonstrated. Dilated intercellular spaces, basal zone hyperplasia, and extracellular matrix deposition are commonly found in EoE biopsies [178,179]. Furthermore, mucosal impedance testing in EoE adults before and after treatment confirms that oesophageal mucosal integrity is significantly impaired in active EoE patients compared to healthy controls and that it inversely correlates, both in vitro and in vivo, with eosinophilia. Mucosal integrity is still impaired in EoE patients in remission compared to healthy controls, suggesting a potential innate barrier defect, this is similar to what happens in other allergic diseases [5] Additionally, histological changes, similar to EoE and EoE transcriptome, can be induced in vitro after stimulation by Th2 cytokines, suggesting the potential role of inflammatory responses in determining barrier dysfunction and eosinophil recruitment [180].

Food allergens and presumably aeroallergens, gain access through a dysfunctional oesophageal barrier and trigger a Th2 inflammatory response, which in turn contributes to disruption of the epithelial barrier, in a self-perpetuating process [181]. Specifically, cytokines secreted by local oesophageal cells promote a Th2-skewing and the presentation of allergens to CD4 T naive cells favours their differentiation to a Th2 phenotype. In particular, epithelial cells secrete TSLP—a “master regulator” of the Th2 response—as it promotes the dendritic cells’ Th2-skewing ability, and basophil maturation [182]. TSLP is overexpressed in EoE biopsies compared to healthy controls [183] and genetic studies have found particular polymorphisms of TSLP and its receptors to be associated with EoE [184]. Furthermore, mouse models have demonstrated that skin sensitisation with food allergens, followed by oral challenges with the same foods, can induce oesophageal lesions similar to EoE, suggesting a possible connection between allergic epicutaneous sensitisation and eosinophilic inflammation of the oesophagus [165].

Activated Th2 cells can amplify an inflammatory response through the secretion of Th2 cytokines, such as IL-4, IL-13, and IL-5, that are increased in both the oesophageal and peripheral blood of active EoE patients [176,185,186].

IL-4 induces the differentiation of naïve Th cells into the Th2 and promote B cell switching to produce IgE [162]. IL-5 is secreted by Th2 cells and eosinophils and favours eosinophil maturation and migration into the oesophageal epithelium [162].

IL-13 has a pivotal role in EoE as it not only contributes to the downregulation of molecules involved in barrier integrity, but it also promotes the recruitment of eosinophils, through the upregulation of eotaxin-3 expression by epithelial cells and of periostin expression by fibroblasts [187]. In particular, eotaxin-3 is encoded by the CCL26 gene and its expression is promoted by IL-13 and IL-4 through Signal Transducer and Activator of Transcription 6 (STAT6) binding to its promoter gene.

Several cells are involved in EoE inflammation. Among them, eosinophils certainly play a key role. They act as antigen presenting cells (APC), enhance inflammatory cascade through production of cytokines (e.g., IL-1, IL-3, IL-4, IL-5, GMCSF, TGFß) and activate inflammatory cells such as T cells, mast-cells, and basophils. They also contribute to tissue remodelling and fibrosis through the secretion of TGFß, eosinophilic peroxidase (EPO) and a major binding protein (MBP) that cause mucosal damage and oesophageal dysmotility [181,188].

Mast-cells and basophils recruited to the oesophagus further enhance inflammatory responses and oesophageal dysmotility, by secreting cytokines such as TGFß1 [183,189,190].

## 11. Therapeutic Implications and Novel Therapeutic Strategies

Conventional management of EoE currently includes proton pump inhibitors (PPI), elimination diets and topical steroids. They all seem to act by targeting the key elements in EoE pathogenesis, the epithelial barrier, the Th2 response and their mutual interaction.

PPIs may act by reducing oesophageal epithelium damage due to acid exposure. However, the PPI response in EoE cannot be merely explained by its well-established antisecretory activity. Indeed, it seems that PPIs may also exert an anti-inflammatory activity, as they can downregulate oesophageal Th2-cytokines and eotaxin-3 [191,192,193,194]. In particular, PPIs block STAT6 binding to the CCL26 promoter inhibiting in vitro eotaxin-3 expression [195].

Various elimination diets have been successfully used in EoE. The Elemental Diet is the most effective, as it induces histological remission in nearly 90% of patients, but multi-food elimination diets may be an alternative choice as they have a lower cost and better palatability [155,196]. Dietary therapy modulates mast cell density and activity, leading to the reduction in mast-cell proteases damaging tissue [183,197].

Ingested topical steroids are effective in obtaining clinical and histological remission in EoE through multiple mechanisms [198,199,200]. Steroid therapy is proven to normalize EoE transcriptome, including IL-13, CCL26, and filaggrin [176,201]. Restoration of filaggrin levels in steroid-treated EoE seems to contribute to restoring epithelial barrier function. Furthermore, steroid therapy seems to have a wider effect on oesophageal tight junctions, leading to a decrease in epithelial spongiosis [202]. Finally, steroids reduce mast-cell recruitment, degranulation, and eosinophil recruitment mediated by IL-5 [185,194]

Notably, some patients are refractory to conventional therapeutic strategies; moreover, compliance to daily medications may be poor, and responses to steroids might be lost over time.

In light of these limitations, biological agents targeting key cytokines, such as IL-4, IL-5 and IL-13, as well as IgE and TNF, have been studied for the treatment of EoE. Some of the studies have successfully progressed to phase III trials (see Table 1)

IL-5 blockage by Mepolizumab and Reslizumab has been suggested as a potential treatment strategy for EoE, since it interferes with eosinophil maturation and migration into the oesophageal epithelium. Indeed, it has also resulted effective in reducing peak and mean oesophageal intraepithelial eosinophil counts, but a significant clinical improvement was not observed in three separate studies in children [203,204,205]. Treatment with Omalizumab, an anti-IgE antibody, failed to induce either reduction in oesophageal and peripheral eosinophilia or clinical improvement [166,206].

IL-13 has been shown to play a crucial role in epithelial barrier integrity, as demonstrated by transcriptional changes of EDC induced by IL-13 itself. By targeting this molecule and its receptor, it could be possible to restore epithelial barrier function, Nevertheless, studies on QAX576, a monoclonal antibody targeting IL-13, have provided scarce results in a small adult cohort. It has been shown to reduce oesophageal eosinophilia and to downregulate important proteins involved in tissue integrity (collagens, periostin, keratins), but no clinical improvement has been noted [207] No studies in children have been conducted yet. Dupilumab, a monoclonal antibody targeting IL-4 receptor alpha chain (IL-4Rα), common to both IL-4 and IL13, appeared to be effective in a phase 2 trial and seems to induce the reduction in Th2 inflammation markers and eosinophilic inflammation, as well as the improvement of endoscopic features. In addition, it seems to be effective in increasing oesophageal distensibility suggesting the potential role of this therapy in reducing tissue damage and consequent remodelling [154]. A phase 3 trial on both the adolescent and adult population is currently enrolling patients.

This evidence strengthens the concept of the epithelial barrier’s central role in EoE pathogenesis and suggests that a targeted therapy, restoring barrier integrity by Th2 inflammation modulation, might be the most promising strategy for EoE.

## 12. Conclusions

A common characteristic of allergic diseases is the impairment of the epithelial barrier, which is skewed toward loss of differentiation, reduced junctional integrity, and altered innate defence.

Despite our growing knowledge of the pivotal role of barrier dysfunction in the initiation of allergic diseases, many important questions regarding mechanisms affecting normal barrier function remain unanswered. Identifying biomarkers for routine practice is essential for developing new strategies and intervention that is targeted towards restoring barrier impairment.
